# Sympathetic and Vagal Nerve Activity in COPD: Pathophysiology, Presumed Determinants and Underappreciated Therapeutic Potential

**DOI:** 10.3389/fphys.2022.919422

**Published:** 2022-06-23

**Authors:** Jens Spiesshoefer, Binaya Regmi, Matteo Maria Ottaviani, Florian Kahles, Alberto Giannoni, Chiara Borrelli, Claudio Passino, Vaughan Macefield, Michael Dreher

**Affiliations:** ^1^ Department of Pneumology and Intensive Care Medicine, University Hospital RWTH Aachen, Aachen, Germany; ^2^ Institute of Life Sciences, Scuola Superiore Sant’Anna, Pisa, Italy; ^3^ Department of Cardiology and Vascular Medicine, University Hospital RWTH Aachen, Aachen, Germany; ^4^ Human Autonomic Neurophysiology Laboratory, Baker Heart and Diabetes Institute, Melbourne, VIC, Australia; ^5^ Department of Anatomy and Physiology, University of Melbourne, Melbourne, VIC, Australia

**Keywords:** sympathetic drive, chronic lung disease, cardiovascular stress, autonomic nervous system, vagal activity

## Abstract

This article explains the comprehensive state of the art assessment of sympathetic (SNA) and vagal nerve activity recordings in humans and highlights the precise mechanisms mediating increased SNA and its corresponding presumed clinical determinants and therapeutic potential in the context of chronic obstructive pulmonary disease (COPD). It is known that patients with COPD exhibit increased muscle sympathetic nerve activity (MSNA), as measured directly using intraneural microelectrodes—the gold standard for evaluation of sympathetic outflow. However, the underlying physiological mechanisms responsible for the sympathoexcitation in COPD and its clinical relevance are less well understood. This may be related to the absence of a systematic approach to measure the increase in sympathetic activity and the lack of a comprehensive approach to assess the underlying mechanisms by which MSNA increases. The nature of sympathoexcitation can be dissected by distinguishing the heart rate increasing properties (heart rate and blood pressure variability) from the vasoconstrictive drive to the peripheral vasculature (measurement of catecholamines and MSNA) (**Graphical Abstract Figure 1**). Invasive assessment of MSNA to the point of single unit recordings with analysis of single postganglionic sympathetic firing, and hence SNA drive to the peripheral vasculature, is the gold standard for quantification of SNA in humans but is only available in a few centres worldwide because it is costly, time consuming and requires a high level of training. A broad picture of the underlying pathophysiological determinants of the increase in sympathetic outflow in COPD can only be determined if a combination of these tools are used. Various factors potentially determine SNA in COPD (**Graphical Abstract Figure 1**): Obstructive sleep apnoea (OSA) is highly prevalent in COPD, and leads to repeated bouts of upper airway obstructions with hypoxemia, causing repetitive arousals. This probably produces ongoing sympathoexcitation in the awake state, likely in the “blue bloater” phenotype, resulting in persistent vasoconstriction. Other variables likely describe a subset of COPD patients with increase of sympathetic drive to the heart, clinically likely in the “pink puffer” phenotype. Pharmacological treatment options of increased SNA in COPD could comprise beta blocker therapy. However, as opposed to systolic heart failure a similar beneficial effect of beta blocker therapy in COPD patients has not been shown. The point is made that although MSNA is undoubtedly increased in COPD (probably independently from concomitant cardiovascular disease), studies designed to determine clinical improvements during specific treatment will only be successful if they include adequate patient selection and translational state of the art assessment of SNA. This would ideally include intraneural recordings of MSNA and—as a future perspective—vagal nerve activity all of which should ideally be assessed both in the upright and in the supine position to also determine baroreflex function.

## GRAPHICAL ABSTRACT

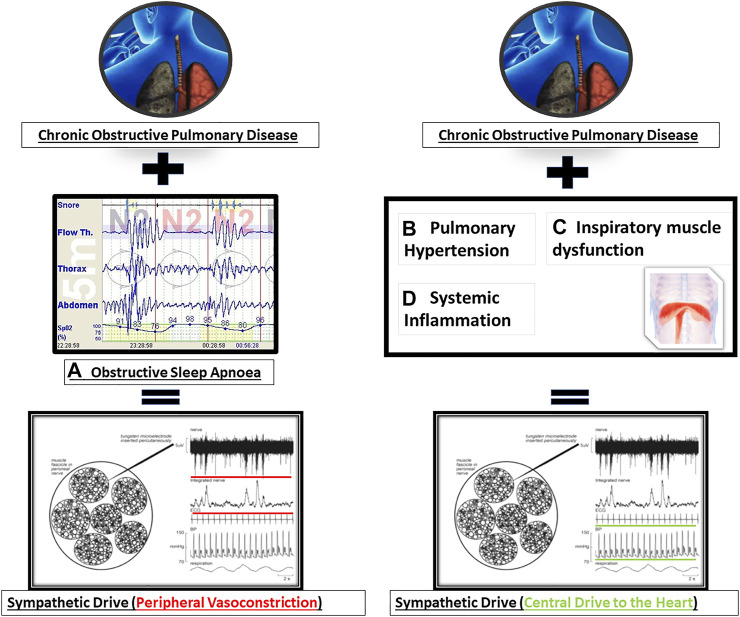

## 1 Background to COPD

Chronic obstructive pulmonary disease (COPD) is a disease characterised by chronic airflow limitation, especially in expiration. Obstruction and hyperinflation alongside systemic inflammation burden the respiratory muscles, especially the diaphragm, which may lead to chronic ventilatory insufficiency with first nocturnal, and later daytime, hypercapnia throughout the disease course (Ntritsos et al., 2018). However, clinically different phenotypes—including hypercapnic variants—of COPD can be encountered, that were traditionally referred to as “pink puffers” with predominantly emphysematous changes to the lung, and “blue bloaters” with predominantly air trapping and hyperinflation (Ntritsos et al., 2018).

According to global prevalence estimates, approximately 9% of men and 6% of women have COPD, making it one of the most prevalent diseases worldwide, and the incidence of COPD is expected to increase further based on population demographics. In both COPD phenotypes, particularly in cases showing overlapping features, the disease has a significant impact and is one of the major causes of death in the developed world (Ntritsos et al., 2018).

Strategies to improve the overall prognosis of patients with COPD are therefore highly desirable. However, although guideline-based therapy with inhaled beta-agonists and anticholinergics improves functional status (and hence the daily symptom burden) in many patients, this pharmacotherapy has not yet been shown to reduce mortality in patients with COPD (Lakshmi et al., 2017; Ntritsos et al., 2018). This makes innovative studies and pathophysiological concepts relating to COPD an extremely important topic.

## 2 The Sympathetic Nerve Activity Axis and Methodology for Its State of the Art Assessment

There are several methods for assessing sympathetic nerve activity (SNA). Heart rate (HR) or blood pressure (BP) variability (HRV and BPV, respectively) provide a more general indication of autonomic imbalance and of increases in the heart rate-increasing central drive properties of SNA in particular (Macefield et al., 2010; Karemaker, 2017).

On the other hand catecholamines and MSNA directly reflect the SNA axis and, as such, the peripheral drive to the vasculature (i.e. vasoconstriction) of the SNA axis which also explains lack of clear correlations between HRV, BPV and MSNA (Notarius et al., 1999; Floras, 2009; Macefield et al., 2010; Karemaker, 2017).

**FIGURE 1:**
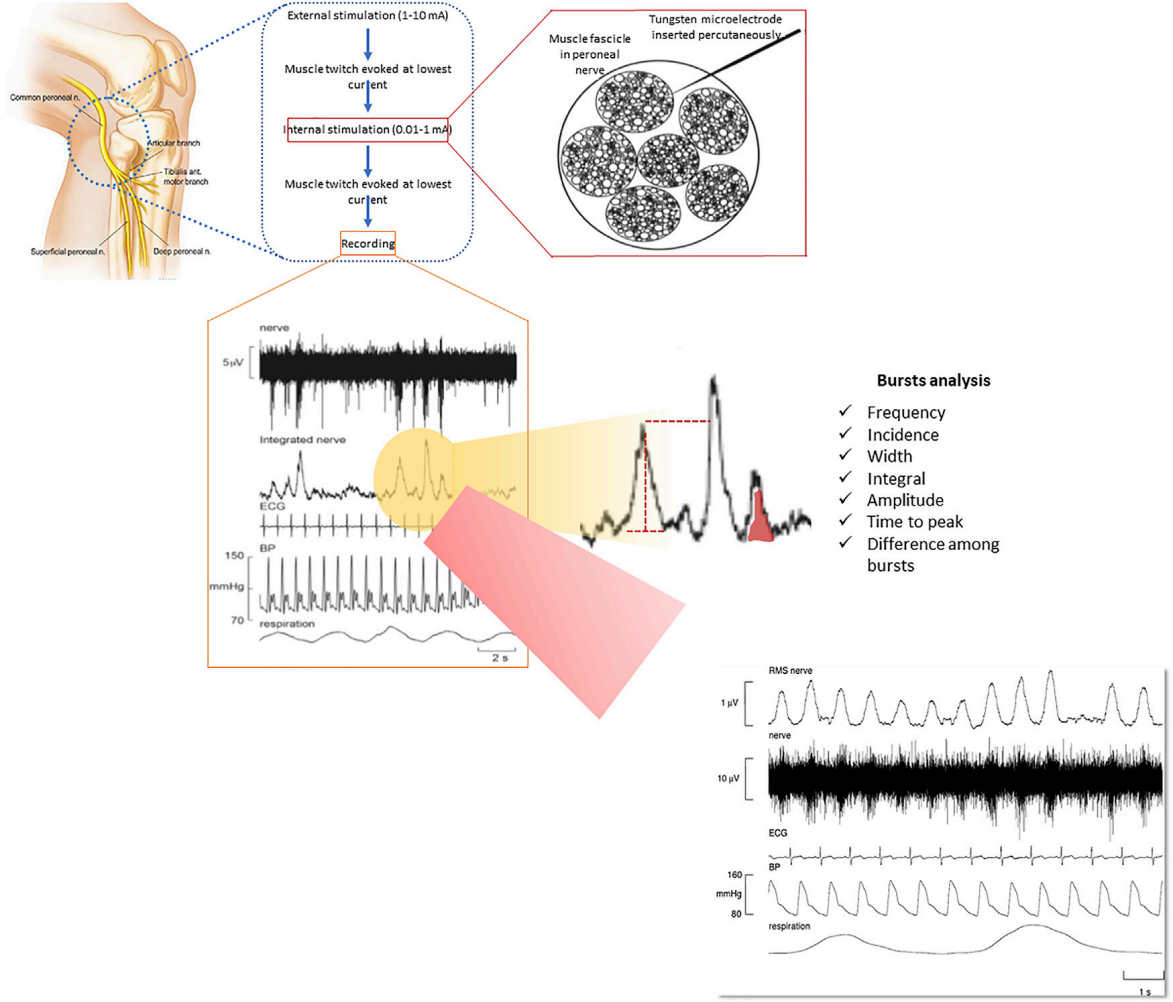
Methodology of recording muscle sympathetic nerve activity (MSNA) through microneurography and its elevation in a patient with COPD. Reproduced, with permission, from Ashley et al. (2010). MSNA is measured via a tungsten microelectrode inserted percutaneously into a muscle fascicle of an accessible peripheral nerve. Various components of the bursts can be measured, providing a comprehensive assessment of muscle vasoconstrictor drive that is put into a broader context of other physiological signals, including heart rate, blood pressure and respiration (see lower part of the figure for representative recordings).

In particular, MSNA recordings allow invasive real-time monitoring of the sympathetic burst rate within the efferent peroneal nerve, which provides a direct measure of SNA (Elam et al., 2002; Ashley et al., 2010; Macefield and Wallin, 2018) (Figure 1). This methodology represents the gold standard for quantification of SNA in humans (peripheral vasoconstriction in particular) but is only available in few centres worldwide because it is costly, time consuming and requires a high level of training (Heindl et al., 2001; Elam et al., 2002; Macefield et al., 2010). Vaughan Macefield recently developed an approach via which MSNA can be recorded from single neurons rather than from multiple units (Heindl et al., 2001; Elam et al., 2002; Macefield et al., 2010; Macefield and Henderson, 2019). This allows in-depth analysis of MSNA in humans to the point of quantifying the firing frequency, firing probability and the extent of multiple firing of single post ganglionic sympathetic neurons (Heindl et al., 2001; Elam et al., 2002; Macefield et al., 2010; Macefield and Henderson, 2019) (Figure 1).

Lack of using a combination of the above mentioned methods in a large enough cohort of patients represents a dilemma because there are methodological limitations if only single metrics of SNA are used. Indeed, using a multimodal approach insights can be generated that facilitate understanding of the nature of increased SNA (“central” heart rate increasing vs peripheral vasoconstriction) in any disease. This also facilitates understanding of the pathophysiological mode of action necessary for any pharmacotherapy to be successful in attacking on increased SNA in various diseases as part of modern precision medicine.

## 3 COPD: Association With Increased Sympathetic Nerve Activity

Increased SNA in COPD has been documented using all the above methods (Keller et al., 1971; Heindl et al., 2001; Elam et al., 2002; Macefield et al., 2010; Chhabra et al., 2015; Mohammed et al., 2015; Goulart et al., 2016; Zangrando et al., 2018). The respiratory and cardiovascular systems are tightly coupled in order to maximise the delivery of oxygen and the removal of carbon dioxide from body tissues. It is therefore not surprising that diseases affecting the respiratory system may have cardiovascular consequences (Macefield, 2012).

Indeed, several studies have documented an increase in MSNA in COPD (Heindl et al., 2001; Raupach et al., 2008; Macefield et al., 2010; Haarmann et al., 2016) (Table 1). A Germany based group was the first to prove increased MSNA in COPD in 2001 in 6 hypoxic COPD patients as compared to matched controls. The point was also made that MSNA decreased following oxygen administration (Heindl et al., 2001). In following experiments it was shown by this group that (in 16 normoxic COPD patients) slow breathing reduces MSNA and that there is an association between increased MSNA and limited exercise capacity in patients with COPD (as shown in 16 COPD patients) (Raupach et al., 2008; Haarmann et al., 2016).

**TABLE 1:** Summary of key studies invasively investigating sympathetic nerve activity (SNA) in chronic obstructive pulmonary disease (COPD).

First Author, year (*Journal*)	Cross Sectional	Longitudinal	Intervention (Subjective)	Mortality Endpoint	Key Finding
Heindl, 2001 (*Am J Respir Crit Care Med*)	**+**	**-**	**-**	**-**	MSNA is increased in COPD
Macefield, 2012 (*Front Physiol*)	**+**	**-**	**-**	**-**	MSNA is increased in COPD; first in human single unit recording showing that multiple firing is increased in COPD (unlike in systolic heart failure)
Andreas, 2014 (*Lung*)	**-**	**+**	**-**	**+**	Increased MSNA in COPD is associated with morbidity and mortality
Haarmann, 2015 (*BMC Pulm Med*)	**-**	**+**	**+**	**-**	Inhaled β-agonist does not modify MSNA in COPD
Haarmann, 2016 (*COPD*)	**+**	**-**	**-**	**-**	Sympathetic activation is associated with exercise limitation in COPD

However, in COPD the background to increased MSNA likely is multifactorial and therefore deserves a multimodal approach to phenotype (i.e. vasoconstrictive as opposed to central drive to the heart SNA increase) and eventually understand it (**Graphical Abstract Figure 1**).

What is even more striking then is the observation that COPD likely is associated with increased MSNA irrespective of (long before the development of, respectively) concomitant cardiovascular disease (Macefield et al., 2010).

Single-unit recordings, which provide richer information than standard multi-unit recordings of MSNA, have revealed that there is increase in central sympathetic drive in COPD as demonstrated in 18 COPD patients (Macefield et al., 2010). Unlike multi-unit recordings single unit recordings allow for insights into the firing behaviour of single postganglionic muscle vasoconstrictor neurons (Macefield et al., 2010). Compared to healthy subjects with low levels of resting MSNA, single-unit recordings revealed that the augmented MSNA seen in COPD, and systolic heart failure (HF) were each associated with an increase in firing probability and mean firing rates of individual neurons (Macefield et al., 2010). However, unlike patients with heart failure, all patients with COPD exhibited an increase in multiple within-burst firing which, it is argued, reflects an increase in central sympathetic drive (Macefield et al., 2010). These observations emphasize the differences by which the sympathetic nervous system grades its output in health and disease, with an increase in firing probability of active neurons (as seen in COPD) and recruitment of additional neurons (as seen in HF) being the dominant mechanisms (Macefield et al., 2010).

A multi-unit recording (including explanations to its measurement) of MSNA from a patient with COPD is shown in Figure 1. In this patient, it can be seen that bursts of MSNA occur with every heartbeat, reflecting a markedly elevated sympathetic outflow. When analyzing MSNA recordings it should be kept in mind that the amplitude of the MSNA bursts is dependent on the position of the needle electrode and it is therefore difficult to obtain comparable inter- and intra-patient recordings (Floras, 2009; Ashley et al., 2010; Macefield et al., 2010; Macefield and Wallin, 2018; Macefield and Henderson, 2019). This can be solved through normalization procedures or advanced processing of MSNA to obtain calibrated MSNA variability series but it remains an important aspect to consider when applying MSNA recording in large scale pathophysiological studies (Floras, 2009; Ashley et al., 2010; Macefield et al., 2010; Macefield and Wallin, 2018; Macefield and Henderson, 2019).

To the best of our knowledge, the SNA axis has not yet been studied in COPD using a combination of the above mentioned methods in a large enough cohort of patients making it impossible to determine the extent and nature plus the clinical mechanisms and phenotypes responsible for the increase in SNA in COPD to date. In fact, the cohorts of patients in whom MSNA was measured never exceeded 18 COPD patients, a multimodal approach of SNA assessment comprising catecholamines, heart rate and blood pressure variability and MSNA combined has never been applied and MSNA has not yet been put into the context of distinct clinical and physiological phenotypes of COPD patients (obstructive sleep apnea, hypercapnia with inspiratory muscle dysfunction, precapillary pulmonary hypertension (PH), increased levels of systemic inflammation all as reviewed later in this review).

## 4 Lessons Learned From Studying Sympathetic Nerve Activity in Cardiovascular Diseases

Cardiovascular diseases and systolic heart failure (HF) in particular, is a disease in which the relationship between increased sympathetic activity and poor clinical outcomes, and as a therapeutic target, has long been established (Guzzetti et al., 1995; Floras, 2003; Mansfield et al., 2003; Spaak et al., 2005; Floras, 2009; Haarmann et al., 2016). Indeed, elevated levels of MSNA are a better known feature of many diseases affecting the cardiovascular system than of respiratory diseases per se (Grassi et al., 1998; Van De Borne et al., 1998; Elam et al., 2002). This comprises either directly or indirectly HF, essential hypertension, pregnancy-induced hypertension, renovascular hypertension and chronic kidney disease (Elam et al., 2002; Floras, 2009).

Mechanisms for the sympathoexcitation differ in each of these pathophysiological states and are generally still not completely understood, but it is clear that the increase in sympathetic outflow is not limited to the muscle vascular bed but affects many organ systems (Notarius et al., 1999; Floras, 2009; Bruno et al., 2012; Grassi et al., 2019). For example, the sympathoexcitation seen in HF is associated with increases in whole-body (plasma) catecholamines, as well as increases in noradrenaline spillover to the heart and kidneys. Indeed, it is the increase in cardiac sympathetic drive that is deleterious in HF, exacerbating as it does the loss of cardiac function and hence providing the rationale for ß-receptor blockade in HF (Floras, 2009; Grassi et al., 2019). In fact, higher sympathetic outflow has been associated with increased morbidity and mortality in systolic HF (Cohn et al., 1984; Latini et al., 2004; Karasulu et al., 2010).

Therefore, currently recommended therapies for HF target elevated sympathetic outflow (Antman et al., 2016; Jessup et al., 2016; Ponikowski et al., 2016; Seferovic et al., 2019). Many of these guideline-recommended HF treatments (most importantly ß-blockers) have been shown to significantly decrease mortality in patients with HF, something that has not consistently been shown for any COPD treatment to date, despite the fact that COPD is one of the most prevalent diseases worldwide (Lakshmi et al., 2017; Ntritsos et al., 2018).

## 5 Rationale and Aim to Focus on Sympathetic Nerve Activity in COPD

Despite several previous observations, the underlying mechanisms for the sympathoexcitation seen in COPD and their relationship with concomitant cardiovascular disease are not completely clear. It is known that sustained hypoxaemia (as seen in COPD) causes a long-lasting increase in MSNA and blood pressure, and that this persists even following the return to normoxia (Morgan et al., 1995; Narkiewicz et al., 1999; Tamisier et al., 2005).

Primary lung damage per se, as seen in COPD, also increases plasma catecholamines, irrespective of concomitant cardiovascular disease, suggesting a general increase in sympathetic outflow (Keller et al., 1971). Consecutive inspiratory muscle dysfunction, precapillary PH and systemic inflammation may also be involved, but this has not been investigated or reviewed to date.

Therefore, increased SNA in COPD could explain why patients with COPD are almost three times more likely to die of HF than smokers not diagnosed with COPD (Huiart et al., 2005). Such data suggest that sympathoexcitation may be involved in the pathophysiology of COPD-related mortality, independent of the development of cardiovascular disease in the same COPD patient (Huiart et al., 2005). However, systematic analyses on the mechanisms involved in the sympathoexcitation occurring in patients with COPD are currently lacking, which provides the rationale for this critical review of existing literature. Owing to decisive gaps in knowledge this includes suggestions for future translational research projects that could help elucidate both the precise mechanisms mediating increased SNA and its corresponding clinical phenotypes.

### 5.1 Obstructive Sleep Apnoea: Overlap With COPD and Impact on Sympathetic Nerve Activity

Obstructive sleep apnoea (OSA) might be one of the most significant determinants of increased MSNA in COPD. Coexisting COPD and OSA is now commonly referred to as “COPD-OSA overlap syndrome” (Kasai et al., 2012; McNicholas, 2018). It is estimated that at least 1 billion people worldwide are affected by OSA, half of whom have moderate to severe (and, therefore, clinically significant) OSA (McNicholas, 2018; Ntritsos et al., 2018; Benjafield et al., 2019). Therefore, the worldwide prevalence of COPD-OSA overlap syndrome in the general population might be as high as 1–2% (McNicholas, 2018).

Despite this, little research has been conducted on the COPD-OSA overlap syndrome (McNicholas, 2018).

The coexistence of COPD and OSA could be associated with particularly high sympathetic outflow, manifested as increased vasoconstriction, that would persist even in the awake state and can be reversed by nocturnal use of continuous positive airway pressure:

This rationale is supported by three arguments.

First, OSA is associated with cyclic oxygen desaturations, and it is known that—as noted above—cyclic hypoxaemia causes a sustained increase in MSNA and BP that persists even after the return to normoxia (Morgan et al., 1995; Tamisier et al., 2005; Macefield, 2012).

Second, it is also known that episodes of airway obstruction during sleep (that can, by definition, be found in OSA patients) cause an increase in MSNA even under normoxic conditions and that this increase also persists in the awake state, leading, in the long term, to the development of arterial hypertension (Morgan et al., 1995; Narkiewicz et al., 1999; Macefield and Elam, 2002; Hansen and Sander, 2003; Tamisier et al., 2005).

Third, OSA is known to have unfavourable effects on sleep architecture, decreasing objective sleep quality and the time spent in deep sleep, all of which adversely impacts on nocturnal MSNA, as recently shown by our group (Spiesshoefer et al., 2019a; Oldenburg and Spiesshoefer, 2020). Alterations in sleep stages with less deep sleep and altered individual chronobiology can potentially cause or promote major cardiovascular diseases through sympathoexcitation, perhaps beyond what might be explained by OSA severity alone (Pagani et al., 1997; Van de Borne et al., 1997; Penzel et al., 2007; Camillo et al., 2011).

Curiously, the clinical phenotype in which such a marked sympathoexcitation with predominantly increased vasoconstriction due to OSA could be encountered, likely corresponds to the phenotype of the “blue bloater” rather than the “pink puffer”, a hypothesis that could be investigated in future experiments.

### 5.2 Lung and Inspiratory Muscle Function as Potential Determinants of Sympathetic Nerve Activity in COPD

Despite being an obstructive lung disease, metrics reflecting inspiratory muscle dysfunction rather than the actual extent of obstruction show the closest association with hypercapnic ventilatory failure in COPD. Inspiratory muscle dysfunction with hypercapnic ventilatory failure in COPD may contribute to an additional increase in sympathetic outflow, but this question has not been addressed to date. In fact, only one recent study showed that maximal inspiratory mouth occlusion pressure (PIMax) is inversely correlated with sympathetic outflow, manifested as a presumed increase in central drive to the heart, as assessed indirectly by heart rate variability, in COPD (Goulart et al., 2016). In line with this, improvements of heart rate variability after exercise training have been documented in COPD patients (Camillo et al., 2011). This makes pulmonary rehabilitation with improvements in muscle strength and exercise capacity a widely available therapeutic approach to reduce increased SNA in COPD.

However, in this study neither inspiratory muscle dysfunction nor MSNA have been directly assessed using gold standard techniques. These would have comprised invasive measurement of the transdiaphragmatic pressure response to supramaximal magnetic cervical phrenic nerve stimulation (CMS) by double balloon catheters and invasive measurement of MSNA by microneurography. Combined application of PIMax, diaphragm ultrasound-derived metrics and cervical phrenic nerve stimulation-derived metrics provides a comprehensive pathophysiological picture of inspiratory muscle function, as evaluated and applied by our group (Spiesshoefer et al., 2019b; Spiesshoefer et al., 2019c; Spiesshoefer et al., 2019d; Spiesshoefer et al., 2020a; Spiesshoefer et al., 2020b).

Clinically, once inspiratory muscle dysfunction is severe enough to result in muscle pump failure, it likely causes nocturnal hypercapnia that then might extend into the awake state in COPD, predisposing to precapillary pulmonary hypertension.

### 5.3 Pulmonary Hypertension as a Potential Contributor to Sympathetic Nerve Activity in COPD

Precapillary PH might develop in COPD, especially in those with advanced disease and respiratory insufficiency with chronic hypoxemia and/or daytime hypercapnia (Simonneau et al., 2013). PH probably further increases sympathetic outflow in COPD. Indeed, there is evidence that PH and increased right atrial pressure (as a surrogate marker of right heart dysfunction) directly increase sympathetic outflow, occurring as an increase in central drive to the heart, as supported by findings from our group (Velez-Roa et al., 2004; Ciarka et al., 2007; Spiesshoefer et al., 2019e).

This, and additional cardiac function and haemodynamic variables, can be assessed using state-of-the-art comprehensive transthoracic echocardiography, combined with a non-invasive hemodynamic monitor, as recently established by our group (Vahanian et al., 2007; Lang et al., 2015; Oldenburg et al., 2015). However, no study has yet assessed the impact of PH and right HF on MSNA in patients with COPD or COPD-OSA overlap syndrome. Notably, PH may have impact on inspiratory muscle function in COPD, and therefore lung and cardiac insufficiency may influence each other (Kabitz et al., 2008; Spiesshoefer et al., 2019f).

In this context, a comprehensive protocol for ultrasound of the diaphragm (the key inspiratory muscle) has recently been established by our group and applied to patients with precapillary PH levels of differing severity based on the 6-min walking distance (6MWD) (Spiesshoefer et al., 2019f; Spiesshoefer et al., 2020a).

### 5.4 Inflammation as a Potential Contributor to Sympathetic Nerve Activity in COPD

Systemic inflammation has been reported to be present in COPD, as shown by increased levels of circulating interleukin (IL)-1 beta, IL-6, tumour necrosis factor-alpha (TNF-α), high-sensitivity C-reactive protein (CRP) and total white blood cell count (Sethi et al., 2012; Barnes, 2013; Chhabra et al., 2015; Okamoto et al., 2015). Levels of these proinflammatory markers most likely increase in response to structural and functional lung function impairment in COPD and impact on inspiratory muscle function as shown by our group in a cohort of patients after lung transplantation (Spiesshoefer et al., 2020c).

Systemic inflammation may have an additive effect on the increase in sympathetic outflow in COPD. Proinflammatory mediators like IL-6 and TNF-α may directly influence inspiratory muscle function, as shown in animal studies (Sethi et al., 2012; Barnes, 2013; Chhabra et al., 2015; Okamoto et al., 2015). Given these proactive effects, these inflammatory markers cannot be considered trivial bystanders of hypoxia, OSA, PH, and inspiratory muscle dysfunction with hypercapnia. Therefore, systemic inflammation, inspiratory muscle function, and potentially sympathetic outflow, may be interrelated in an exponential rather than linear fashion (Spiesshoefer et al., 2019g). Such interrelation has recently been suggested by Chhabra and colleagues, who recently showed a positive correlation between serum IL-6 and the low frequency (0.04–0.15 Hz)/high frequency (0.15–0.40) component ratio of heart rate variability (LF/HF ratio), a marker of sympathetic activation, in patients with COPD (although such an interpretation needs to be made with caution) (Chhabra et al., 2015). Conversely, it has also been shown that inappropriate sympathetic activation may upregulate IL-6 levels (Okamoto et al., 2015).

## 6 Pharmacological Treatment of Increased Sympathetic Nerve Activity in COPD

Certainly, given the evidence for an increase in plasma noradrenaline (but not adrenaline), MSNA and “sympathovagal” imbalance in COPD, there is a rationale for pursuing pharmacological means of reducing sympathoexcitation in COPD, such as through the administration of ß1-blockers to protect the heart (Henriksen et al., 1980; Lipworth et al., 2016). However, there have been few systematic studies examining this issue.

The *New England Journal of Medicine* recently reported the results of the first large prospective randomised placebo-controlled trial investigating the effects of a ß-blocker on exacerbations in patients with COPD (Dransfield et al., 2019). In this study, patients aged 40–85 years were randomised to receive either a ß-blocker (extended-release metoprolol, a ß1-antagonist) or placebo (Dransfield et al., 2019). All patients had a clinical history of COPD, with moderate airflow limitation, and were at increased risk of exacerbations, but without an indication for ß-blocker therapy for cardiovascular comorbidities; patients already taking a ß-blocker or with an established indication for these agents were excluded (Dransfield et al., 2019). The trial was designed to address the hypothesis that increased SNA could be linked to COPD-associated lung damage and that reductions in SNA during treatment with a ß-blocker may translate into decreased time to exacerbation and a reduction in exacerbation severity (Dransfield et al., 2019). However, the outcomes of the trial were negative, with a similar time to first COPD exacerbation in the metoprolol and placebo groups (Dransfield et al., 2019).

This result highlights the need for better insights into increased SNA in COPD, allowing identification of a subgroup of COPD patients who might benefit from ß-blocker therapy. Indeed, all patients in this trial were not stratified for factors indicating a potential benefit of ß-blocker therapy, including an increase in sympathetic outflow. In fact, not a single metric of increased sympathetic activity in the COPD patients was specifically assessed at baseline. Clearly, insights into the features and underlying mechanisms, including defining specific clinical phenotypes and measuring a range of physiological variables, are urgently needed to identify patients who may potentially benefit from individualised therapy to reduce sympathoexcitaiton. It therefore likely is the quantification of the single patient cardiovascular and respiratory autonomic profile, possibly obtained by the maximum precision and rigor by using direct recording techniques and the complex analyses of cardiovascular and respiratory coupling, that will maybe help to identify the target COPD population to assess the benefit of beta-blocker therapy in.

Of course, there is also a rationale for locally *increasing* sympathetic outflow in COPD—specifically to the airways—to improve lung function, but would this have adverse effects elsewhere? A recent study showed that inhaled salmeterol, a long-acting ß_2_-receptor agonist, improved lung function in COPD and caused a small but significant increase in heart rate, but did not affect MSNA (Haarmann et al., 2015).

For successful therapy that targets an increase in cardiac sympathetic drive in COPD, such as the use of a ß-blocker, it would be essential to identify a specific patient phenotype (by physiological and/or clinical variables) characterised by an increase in central drive to the heart, and not just as an increase in MSNA and peripheral vasoconstriction. Curiously, clinically that would likely correspond to the “pink puffer” without OSA and hence predominantly peripheral vasoconstriction, rather than the “blue bloater” phenotype.

Thus, using a comprehensive, multimodal approach and state-of-the-art technology, future research projects must be designed to determine the extent and nature, plus the clinical mechanisms, responsible for the increase in sympathetic outflow in COPD, and their specific targets.

Based on pathophysiological considerations and a small number of previous studies, it can be hypothesised that in COPD patients without an established cardiovascular disease (ideally including age-, sex- and body mass index [BMI]-matched healthy controls) it is concomitant OSA and poor sleep that is independently associated with increased SNA (manifesting as increased drive to the peripheral vasculature with vasoconstriction). Factors including PH**,** inspiratory muscle dysfunction and systemic inflammation would then likely describe a COPD phenotype characterised by increased SNA manifesting an increased central drive to the heart. It is this subset of COPD patients (presumably without OSA) that needs to be specifically identified and enrolled as part of modern precision medicine trials in COPD as a crucial step towards achieving a therapeutic benefit of ß-blocker therapy on exacerbations in COPD.

## 7 The Novel Field of Changes in Parasympathetic Nerve Activity in COPD

While much discussion has been directed towards the sympathoexcitation seen in COPD, we should not forget that the primary neural control of airway diameter is achieved via the parasympathetic nervous system (Stewart et al., 1991; Mohammed et al., 2015; Mohammed et al., 2017; Zangrando et al., 2018). Although circulating hormones and blood gases affect bronchiolar diameter, parasympathetic axons travelling in the vagus nerve supplying the smooth muscle (and glands) of the airways are important in health and disease: bronchomotor neurones are the primary means by which airflow is controlled because there is negligible sympathetic innervation of the airways in humans (van der Beek et al., 2011; Karemaker, 2017).

**FIGURE 2:**
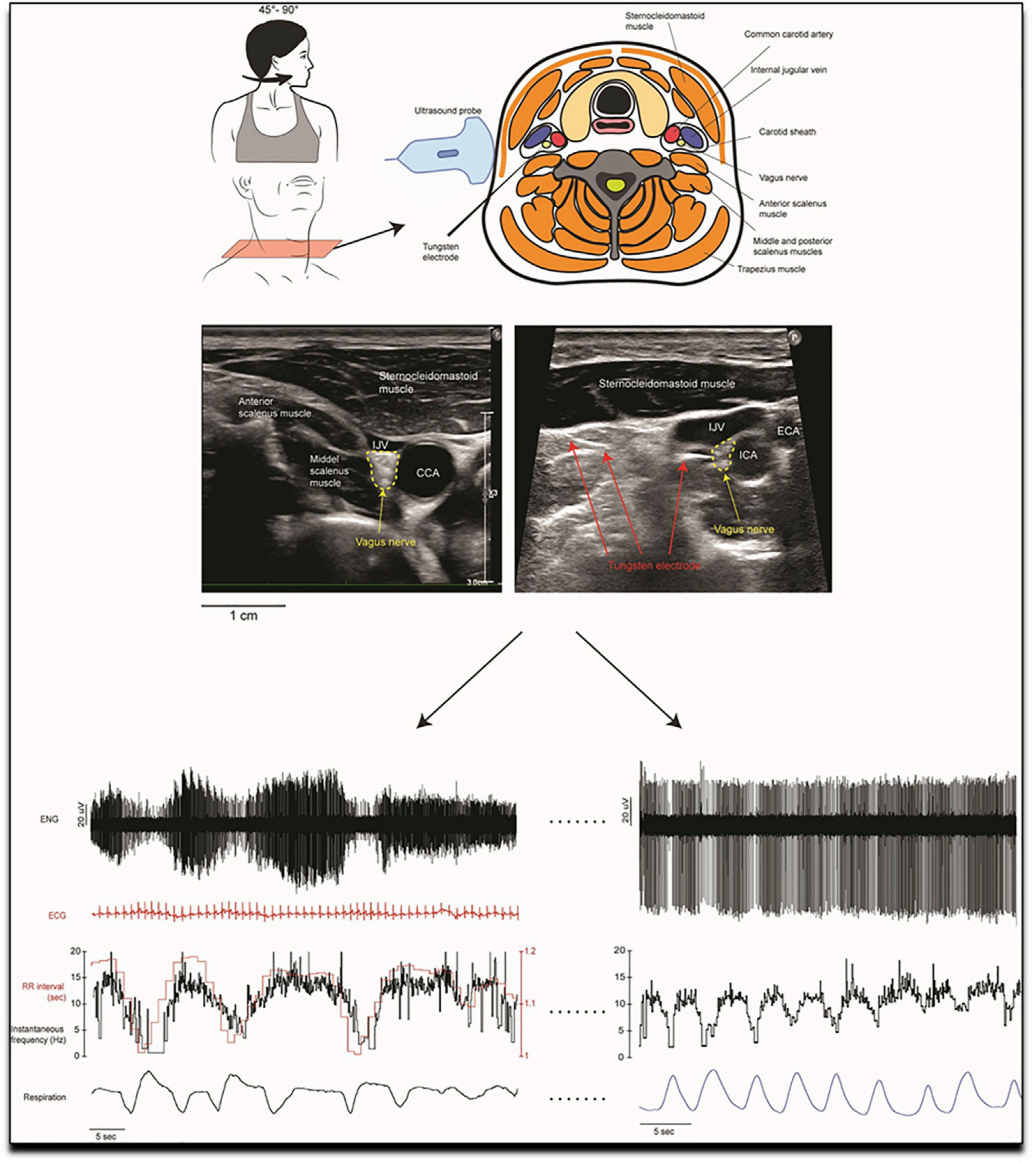
Methodology of the first in human invasive measurement of vagal nerve activity. A tungsten microelectrode is carefully and under ultrasound guidance placed in the vagal nerve, where its activity can then be recorded and analysed. Invasive measurement of vagal nerve activity have not been performed in COPD to date. Reproduced, with permission, from Ottaviani et al. (2020) (Ottaviani et al., 2020). Upper part of the figure: Schematic representation of the orientation of the head, the structures in the neck and the dorsolateral approach of the microelectrode as it is advanced manually towards the right vagus nerve. This approach avoided the carotid artery and jugular vein. The nerve is highlighted in the ultrasound images from two recording sessions which shows the common carotid artery (CCA), internal carotid artery (ICA), external carotid artery (ECA) and the internal jugular vein (IJV) and nearby muscles. The image on the right shows the microelectrode tip as it impales the vagus nerve. Lower part of the figure: Examples of microelectrode recordings from the left cervical vagus nerve in one participant. Lower left recording: The firing of the tonically active unit is covaried with cardiac interval. Variations in spike amplitude reflect slight movements of the microelectrode with neck movements. Superimposed spikes confirm that this was a single-unit recording. Lower right recording: a tonically firing axon, the firing rate of which decreased during inspiration.

In COPD a new trial aims to selectively denervate parasympathetic supply to the airways (Slebos et al., 2019). The point should be made here that selectively denervating parasympathetic supply to the airways is the endpoint here (in an attempt to selectively increase airway diameter, hence decrease the extent of obstruction and hence potentially indirectly decrease SNA overall through improved ventilation and exercise tolerance) and not decreasing parasympathetic drive overall. The background to this is that, clearly, decreased SNA and increased parasympathetic nerve activity should be the pathophysiologically desired endpoints to improve exercise intolerance and potentially mortality in COPD patients. Until recently, the parasympathetic nervous system could only be assessed indirectly in humans, largely through measurement of changes in heart rate variability. However, we now have the means of recording from parasympathetic axons directly, by inserting a microelectrode into the cervical vagus nerve under ultrasound guidance (displayed and explained in greater depth in Figure 2) (Ottaviani et al., 2020).

Respiratory and cardiac modulation of multi-unit recordings of vagal activity has recently been quantified (Patros et al., 2022). With few exceptions, the peak of respiratory modulation coincided with the peak of inspiration, whereas the latencies for the peak in cardiac modulation of vagal activity showed a bimodal distribution: some had peak latencies that preceded the synchronising R-wave whereas others had latencies that followed the R-wave (Patros et al., 2022). Unitary recordings from the human vagus nerve will also help us understand how disease affects the behavior of afferents coming from the heart and great vessels, and from the lungs and airways. We propose making parallel recordings from the cervical vagus nerve and from the common peroneal nerve when participants lay on a tilt table, and taking them from the horizontal to near-vertical position; we have success in obtaining stable recordings of muscle sympathetic nerve activity from the leg when tilting participants, and have no doubt about the stability of the vagus nerve recording during this challenge (Ottaviani et al., 2020). However, given the challenges to obtain (mainly in having the needle in the right position) and analyse (see complexity in correlating vagal activity depicted above) these recordings, those measurements will, for now, likely be available in few dedicated expert centres only.

Curiously, inappropriate sympathetic activation was shown to potentially upregulate IL-6 levels and vagal stimulation was suggested as a possible tool to reduce systemic inflammation, in a different pathological population characterized by increased sympathetic modulation—a “positive loop” that hence may also provide a potential novel treatment pathway in COPD (Diedrich et al., 2021).

This will allow us to assess the firing properties of individual preganglionic parasympathetic axons directed to the airways, as well as to the heart, in the same way we have been able to record from single postganglionic sympathetic axons in awake humans (Macefield and Wallin, 2018).

## 8 Testing Baroreceptor Function to Obtain a Comprehensive Picture of Sympathetic and Parasympathetic Nerve Activity in Humans

It is baroreflex-mediated modifications of the sympathetic drive that are responsible for the changes in heart rate, and they partially explain the link between MSNA and heart rate variations (Marchi et al., 2016; Barbic et al., 2019). Indeed, the link between MSNA and arterial pressure (AP) is widely accepted with MSNA burst rate increasing when blood pressure falls and MSNA becoming silent when blood pressure rises as a result of an operating baroreflex that inhibits sympathetic drive (Marchi et al., 2016; Barbic et al., 2019; Furlan et al., 2019). Therefore, to obtain a comprehensive picture of sympathetic nerve activity in humans tilt-table testing is used to unload the low-pressure baroreceptors, emulating the changes on standing. Head-up tilt causes an increase in MSNA to prevent venous pooling and maintain blood pressure (Marchi et al., 2016; Barbic et al., 2019; Furlan et al., 2019). Therefore, such test can determine whether MSNA and heart rate response and hence baroreceptor function is adequate (Marchi et al., 2016; Barbic et al., 2019; Furlan et al., 2019). Until recently only heart rate variability (HRV) has been used, in which spectral analysis separates the signal into a low frequency (0.04–0.15 Hz) and/high frequency (0.15–0.40 Hz) component. The LF/HF ratio has been referred to as sympathovagal balance, with an increase in LF/HF being attributed to an increase in cardiac sympathetic activation drive, although such an interpretation needs to be made with caution (Barbic et al., 2019; Patros et al., 2022). Recently, variability has also been applied to MSNA bursts, with the resulting calibrated MSNA being more suitable in describing sympathetic control in humans than traditional uncalibrated MSNA (Marchi et al., 2016; Barbic et al., 2019). The latter takes into account absolute values for MSNA bursts, which show greater inter- and intra-individual reproducibility, with the LF component of calibrated but not of uncalibrated MSNA being positively related to tilt angle and with the LF power of heart rate and blood pressure (Marchi et al., 2016; Barbic et al., 2019).

As far as heart rate variability analysis is concerned it should be kept in mind that the newer non-linear analyses (symbolic, dynamic and complexity) could be useful in these patients (Serrão et al., 2020).

We propose making parallel recordings from the cervical vagus nerve and from the common peroneal nerve when participants lay on a tilt table, and taking them from the horizontal to near-vertical position; we have success in obtaining stable recordings of muscle sympathetic nerve activity from the leg when tilting participants, and have no doubt about the stability of the vagus nerve recording during this challenge. This will help us, for the first time in humans, to answer the question as to how the firing properties of atrial receptors or baroreceptors from the aortic arch change with changes in gravitational load, how they change in hypertension and systolic heart failure and COPD and how they affect heart rate, blood pressure and sympathetic outflow to the muscle vascular bed.

## 9 Conclusion

Little is currently known about the underlying pathophysiological mechanisms leading to the sympathoexcitation seen in COPD, especially in COPD-OSA overlap syndrome.

Attempts to better understand these mechanisms require a multimodal, systematic approach to assess both the increase in sympathetic outflow, and to specific target organs, and the presumed underlying clinical mechanisms.

A multimodal, systematic approach should comprise heart rate variability, blood pressure variability, plasma catecholamines and direct recordings of MSNA together to distinguish between differences in sympathetic outflow to the heart and to the muscle vascular bed. Furthermore, the presumed underlying clinical manifestations to assess are OSA and sleep, PH, inspiratory muscle dysfunction and systemic inflammation.

Based on currently available scientific evidence, it likely is OSA with poor sleep that, in the presence of COPD, is independently associated with increased sympathetic Outflow. This is then associated with increased drive to the peripheral vasculature with vasoconstriction. Other clinical variables, namely PH, inspiratory muscle dysfunction and systemic inflammation, may describe additional phenotypes of COPD patients with increased sympathetic outflow to the heart.

By phenotyping COPD patients with regard to the target organs affected by an increase in sympathetic outflow we might define a subgroup that can be enrolled in clinical trials to assess directed pharmacologic treatment. This would be a step towards modern precision medicine in COPD, and a crucial step towards determining the potential therapeutic benefit of ß-blockers on the rate of exacerbations in COPD.

Finally, while much discussion has been directed towards the sympathoexcitation seen in COPD, we should not forget that the primary neural control of airway diameter is achieved via the parasympathetic nervous system. Only now are we invasively assessing the firing properties of individual preganglionic parasympathetic axons directed to the airways, as well as to the heart, in the same way we have been able to record from single postganglionic sympathetic axons in awake humans, more insights into which offer unprecedented future therapeutic potential in COPD.

## References

[B1] Antman E. M. Bax J. Chazal R. A. Creager M. A. Filippatos G. Halperin J. L. (2016). Updated Clinical Practice Guidelines on Heart Failure: An International Alignment. Eur. Heart J. 37, 2096. 10.1093/eurheartj/ehw219 27206818

[B2] Ashley C. Burton D. Sverrisdottir Y. B. Sander M. McKenzie D. K. Macefield V. G. (2010). Firing Probability and Mean Firing Rates of Human Muscle Vasoconstrictor Neurones Are Elevated during Chronic Asphyxia. J. Physiol. 588, 701–712. 10.1113/jphysiol.2009.185348 20051493 PMC2828141

[B3] Barbic F. Heusser K. Minonzio M. Shiffer D. Cairo B. Tank J. (2019). Effects of Prolonged Head-Down Bed Rest on Cardiac and Vascular Baroreceptor Modulation and Orthostatic Tolerance in Healthy Individuals. Front. Physiol. 10. 10.3389/fphys.2019.01061 PMC6716544 31507438

[B4] Barnes P. J. (2013). New Anti-inflammatory Targets for Chronic Obstructive Pulmonary Disease. Nat. Rev. Drug Discov. 12, 543–559. 10.1038/nrd4025 23977698

[B5] Benjafield A. V. Ayas N. T. Eastwood P. R. Heinzer R. Ip M. S. M. Morrell M. J. (2019). Estimation of the Global Prevalence and Burden of Obstructive Sleep Apnoea: a Literature-Based Analysis. Lancet Respir. Med. 7, 687–698. 10.1016/s2213-2600(19)30198-5 31300334 PMC7007763

[B6] Bruno R. M. Ghiadoni L. Seravalle G. Dell'oro R. Taddei S. Grassi G. (2012). Sympathetic Regulation of Vascular Function in Health and Disease. Front. Physiol. 3, 284. 10.3389/fphys.2012.00284 22934037 PMC3429057

[B7] Camillo C. A. Laburu V. d. M. Gonçalves N. S. Cavalheri V. Tomasi F. P. Hernandes N. A. (2011). Improvement of Heart Rate Variability after Exercise Training and its Predictors in COPD. Respir. Med. 105, 1054–1062. 10.1016/j.rmed.2011.01.014 21342757

[B8] Chhabra S. K. Gupta M. Ramaswamy S. Dash D. J. Bansal V. Deepak K. K. (2015). Cardiac Sympathetic Dominance and Systemic Inflammation in COPD. J. Chronic Obstr. Pulm. Dis. 12, 552–559. 10.3109/15412555.2014.974743 25495489

[B9] Ciarka A. Vachièry J.-L. Houssière A. Gujic M. Stoupel E. Velez-Roa S. (2007). Atrial Septostomy Decreases Sympathetic Overactivity in Pulmonary Arterial Hypertension. Chest 131, 1831–1837. 10.1378/chest.06-2903 17400672

[B10] Cohn J. N. Levine T. B. Olivari M. T. Garberg V. Lura D. Francis G. S. (1984). Plasma Norepinephrine as a Guide to Prognosis in Patients with Chronic Congestive Heart Failure. N. Engl. J. Med. 311, 819–823. 10.1056/nejm198409273111303 6382011

[B11] Diedrich A. Urechie V. Shiffer D. Rigo S. Minonzio M. Cairo B. (2021). Transdermal Auricular Vagus Stimulation for the Treatment of Postural Tachycardia Syndrome. Aut. Neurosci. 236, 102886. 10.1016/j.autneu.2021.102886 PMC8939715 34634682

[B12] Dransfield M. T. Voelker H. Bhatt S. P. Brenner K. Casaburi R. Come C. E. (2019). Metoprolol for the Prevention of Acute Exacerbations of COPD. N. Engl. J. Med. 381, 2304–2314. 10.1056/nejmoa1908142 31633896 PMC7416529

[B13] Elam M. McKenzie D. Macefield V. (2002). Mechanisms of Sympathoexcitation: Single-Unit Analysis of Muscle Vasoconstrictor Neurons in Awake OSAS Subjects. J. Appl. Physiology 93, 297–303. 10.1152/japplphysiol.00899.2001 12070217

[B14] Floras J. S. (2003). Sympathetic Activation in Human Heart Failure: Diverse Mechanisms, Therapeutic Opportunities. Acta Physiol. Scand. 177, 391–398. 10.1046/j.1365-201x.2003.01087.x 12609011

[B15] Floras J. S. (2009). Sympathetic Nervous System Activation in Human Heart Failure. J. Am. Coll. Cardiol. 54, 375–385. 10.1016/j.jacc.2009.03.061 19628111

[B16] Furlan R. Heusser K. Minonzio M. Shiffer D. Cairo B. Tank J. (2019). Cardiac and Vascular Sympathetic Baroreflex Control during Orthostatic Pre-syncope. J. Clin. Med. 8, 1434. 10.3390/jcm8091434 PMC6781174 31510103

[B17] Goulart C. d. l. Cristiano Simon J. De Borba Schneiders P. Antunes San Martin E. Cabiddu R. Borghi-silva A. (2016). Respiratory Muscle Strength Effect on Linear and Nonlinear Heart Rate Variability Parameters in COPD Patients. Int. J. Chron. Obstruct Pulmon Dis. 11, 1671–1677. 10.2147/copd.s108860 27555757 PMC4968685

[B18] Grassi G. Cattaneo B. M. Seravalle G. Lanfranchi A. Mancia G. (1998). Baroreflex Control of Sympathetic Nerve Activity in Essential and Secondary Hypertension. Hypertension 31, 68–72. 10.1161/01.hyp.31.1.68 9449393

[B19] Grassi G. D’Arrigo G. Pisano A. Bolignano D. Mallamaci F. Dell’Oro R. (2019). Sympathetic Neural Overdrive in Congestive Heart Failure and its Correlates. J. Hypertens. 37, 1746–1756. 10.1097/hjh.0000000000002093 30950979

[B20] Guzzetti S. Cogliati C. Turiel M. Crema C. Lombardi F. Malliani A. (1995). Sympathetic Predominance Followed by Functional Denervation in the Progression of Chronic Heart Failure. Eur. Heart J. 16, 1100–1107. 10.1093/oxfordjournals.eurheartj.a061053 8665972

[B21] Haarmann H. Mohrlang C. Tschiesner U. Rubin D. B. Bornemann T. Rüter K. (2015). Inhaled β-agonist Does Not Modify Sympathetic Activity in Patients with COPD. BMC Pulm. Med. 15, 46–10. 10.1186/s12890-015-0054-7 25924990 PMC4460951

[B22] Haarmann H. Folle J. Nguyen X. P. Herrmann P. Heusser K. Hasenfuß G. (2016). Sympathetic Activation Is Associated with Exercise Limitation in COPD. J. Chronic Obstr. Pulm. Dis. 13, 589–594. 10.3109/15412555.2015.1136272 26829234

[B23] Hansen J. Sander M. (2003). Sympathetic Neural Overactivity in Healthy Humans after Prolonged Exposure to Hypobaric Hypoxia. J. Physiology 546, 921–929. 10.1113/jphysiol.2002.031765 PMC2342582 12563015

[B24] Heindl S. Lehnert M. Criée C.-P. Hasenfuss G. Andreas S. (2001). Marked Sympathetic Activation in Patients with Chronic Respiratory Failure. Am. J. Respir. Crit. Care Med. 164, 597–601. 10.1164/ajrccm.164.4.2007085 11520722

[B25] Henriksen J. H. Christensen N. J. Kok-Jensen A. Christiansen I. (1980). Increased Plasma Noradrenaline Concentration in Patients with Chronic Obstructive Lung Disease: Relation to Haemodynamics and Blood Gases. Scand. J. Clin. Laboratory Investigation 40, 419–427. 10.3109/00365518009101864 6777857

[B26] Huiart L. Ernst P. Suissa S. (2005). Cardiovascular Morbidity and Mortality in COPD. Chest 128, 2640–2646. 10.1378/chest.128.4.2640 16236937

[B27] Jessup M. Marwick T. H. Ponikowski P. Voors A. A. Yancy C. W. (2016). 2016 ESC and ACC/AHA/HFSA Heart Failure Guideline Update - what Is New and Why Is it Important? Nat. Rev. Cardiol. 13, 623–628. 10.1038/nrcardio.2016.134 27625120

[B28] Kabitz H.-J. Schwoerer A. Bremer H.-C. Sonntag F. Walterspacher S. Walker D. (2008). Impairment of Respiratory Muscle Function in Pulmonary Hypertension. Eur. Respir. J. 114, 165–171. 10.1042/cs20070238 17764445

[B29] Karasulu L. Epöztürk P. Ö. Sökücü S. N. Dalar L. Altın S. (2010). Improving Heart Rate Variability in Sleep Apnea Patients: Differences in Treatment with Auto-Titrating Positive Airway Pressure (APAP) versus Conventional CPAP. Lung 188, 315–320. 10.1007/s00408-010-9237-4 20373105

[B30] Karemaker J. M. (2017). An Introduction into Autonomic Nervous Function. Physiol. Meas. 38, R89–R118. 10.1088/1361-6579/aa6782 28304283

[B31] Kasai T. Floras J. S. Bradley D. (2012). Sleep Apnea and Cardiovascular Disease: a Bidirectional Relationship. Circulation 126, 1495–1510. 10.1161/CIRCULATIONAHA.111.070813 22988046

[B32] Keller R. Lohmann F. W. Schüren K. P. (1971). Catecholamines in Chronic Respiratory Insufficiency. Respiration 28, 273–279. 10.1159/000192843 4938296

[B33] Lakshmi S. Reddy A. Reddy R. (2017). Emerging Pharmaceutical Therapies for COPD. Copd 12, 2141–2156. 10.2147/copd.s121416 PMC5531723 28790817

[B34] Lang R. M. Badano L. P. Mor-avi V. Afilalo J. Armstrong A. Ernande L. (2015). Recommendations for Cardiac Chamber Quantification by Echocardiography in Adults: An Update from the American Society of Echocardiography and the European Association of Cardiovascular Imaging. J. Am. Soc. Echocardiogr. 28, 1–39. e14. 10.1016/j.echo.2014.10.003 25559473

[B35] Latini R. Masson S. Anand I. Monica S. Allen H. Dianne J. (2004). The Comparative Prognostic Value of Plasma Neurohormones at Baseline in Patients with Heart Failure Enrolled in Val-HeFT. Eur. Heart J. 25, 292–299. 10.1016/j.ehj.2003.10.030 14984917

[B36] Lipworth B. Wedzicha J. Devereux G. Vestbo J. Dransfield M. T. (2016). Beta-blockers in COPD: Time for Reappraisal. Eur. Respir. J. 48, 880–888. 10.1183/13993003.01847-2015 27390282

[B37] Macefield V. G. Wallin B. G. Haarmann H. (2010). Firing Patterns of Muscle Vasoconstrictor Neurons in Respiratory Disease. J. Physiol. 588, 925–931. 10.3389/fphys.2012.00153 PMC3358712 22654767

[B38] Macefield V. G. Elam M. (2002). Prolonged Surges of Baroreflex-Resistant Muscle Sympathetic Drive during Periodic Breathing. Clin. Aut. Res. 12, 165–169. 10.1007/s10286-002-0032-z 12269547

[B39] Macefield V. G. (2012). Firing Patterns of Muscle Vasoconstrictor Neurons in Respiratory Disease. Front. Physio. 3, 153–159. 10.3389/fphys.2012.00153 PMC3358712 22654767

[B40] Macefield V. G. Henderson L. A. (2019). Identification of the Human Sympathetic Connectome Involved in Blood Pressure Regulation. Neuroimage 202, 116119. 10.1016/j.neuroimage.2019.116119 31446130

[B41] Macefield V. G. Wallin B. G. (2018). Physiological and Pathophysiological Firing Properties of Single Postganglionic Sympathetic Neurons in Humans. J. Neurophysiology 119, 944–956. 10.1152/jn.00004.2017 29142091

[B42] Mansfield D. Kaye D. M. Brunner La Rocca H. Solin P. Esler M. D. Naughton M. T. (2003). Raised Sympathetic Nerve Activity in Heart Failure and Central Sleep Apnea Is Due to Heart Failure Severity. Circulation 107, 1396–1400. 10.1161/01.cir.0000056520.17353.4f 12642360

[B43] Marchi A. Bari V. De Maria B. Esler M. Lambert E. Baumert M. (2016). Calibrated Variability of Muscle Sympathetic Nerve Activity during Graded Head-Up Tilt in Humans and its Link with Noradrenaline Data and Cardiovascular Rhythms. Am. J. Physiology-Regulatory, Integr. Comp. Physiology 310, R1134–R1143. 10.1152/ajpregu.00541.2015 27009053

[B44] McNicholas W. T. (2018). Comorbid Obstructive Sleep Apnoea and Chronic Obstructive Pulmonary Disease and the Risk of Cardiovascular Disease. J. Thorac. Dis. 10, S4253–S4261. 10.21037/jtd.2018.10.117 30687541 PMC6321895

[B45] Mohammed J. Da Silva H. Van Oosterwijck J. Calders P. (2017). Effect of Respiratory Rehabilitation Techniques on the Autonomic Function in Patients with Chronic Obstructive Pulmonary Disease: A Systematic Review. Chron. Respir. Dis. 14, 217–230. 10.1177/1479972316680844 28774205 PMC5720228

[B46] Mohammed J. Meeus M. Derom E. Da Silva H. Calders P. (2015). Evidence for Autonomic Function and its Influencing Factors in Subjects with COPD: A Systematic Review. Respir. Care 60, 1841–1851. 10.4187/respcare.04174 26487747

[B47] Morgan B. J. Crabtree D. C. Palta M. Skatrud J. B. (1995). Combined Hypoxia and Hypercapnia Evokes Long-Lasting Sympathetic Activation in Humans. J. Appl. Physiology 79, 205–213. 10.1152/jappl.1995.79.1.205 7559221

[B48] Narkiewicz K. Kato M. Phillips B. G. Pesek C. A. Davison D. E. Somers V. K. (1999). Nocturnal Continuous Positive Airway Pressure Decreases Daytime Sympathetic Traffic in Obstructive Sleep Apnea. Circulation 100, 2332–2335. 10.1161/01.cir.100.23.2332 10587337

[B49] Notarius C. F. Butler G. C. Ando S.-i. Pollard M. J. Senn B. L. Floras J. S. (1999). Dissociation between Microneurographic and Heart Rate Variability Estimates of Sympathetic Tone in Normal Subjects and Patients with Heart Failure. Clin. Sci. 96, 557–565. 10.1042/cs19980347 10334961

[B50] Ntritsos G. Franek J. Belbasis L. Christou M. A. Markozannes G. Altman P. (2018). Gender-specific Estimates of COPD Prevalence: A Systematic Review and Meta-Analysis. Copd 13, 1507–1514. 10.2147/copd.s146390 PMC5953270 29785100

[B51] Okamoto L. E. Raj S. R. Gamboa A. Shibao C. A. Arnold A. C. Garland E. M. (2015). Sympathetic Activation Is Associated with Increased IL-6, but Not CRP in the Absence of Obesity: Lessons from Postural Tachycardia Syndrome and Obesity. Am. J. Physiology-Heart Circulatory Physiology 309, H2098–H2107. 10.1152/ajpheart.00409.2015 PMC4698423 26453329

[B52] Oldenburg O. Spiesshoefer J. (2020). Impact of Lifestyle on Sleep. J. Am. Coll. Cardiol. 75, 1000–1002. 10.1016/j.jacc.2019.12.055 32138958

[B53] Oldenburg O. Spießhöfer J. Fox H. Bitter T. Horstkotte D. (2015). Cheyne-Stokes Respiration in Heart Failure: Friend or Foe? Hemodynamic Effects of Hyperventilation in Heart Failure Patients and Healthy Volunteers. Clin. Res. Cardiol. 104, 328–333. 10.1007/s00392-014-0784-1 25373383

[B54] Ottaviani M. M. Wright L. Dawood T. Macefield V. G. (2020). *In Vivo* recordings from the Human Vagus Nerve Using Ultrasound‐guided Microneurography. J. Physiol. 598, 3569–3576. 10.1113/jp280077 32538473

[B55] Pagani M. Montano N. Porta A. Malliani A. Abboud F. M. Birkett C. (1997). Relationship between Spectral Components of Cardiovascular Variabilities and Direct Measures of Muscle Sympathetic Nerve Activity in Humans. Circulation 95, 1441–1448. 10.1161/01.cir.95.6.1441 9118511

[B56] Patros M. Ottaviani M. M. Wright L. Dawood T. Macefield V. G. (2022). Quantification of Cardiac and Respiratory Modulation of Axonal Activity in the Human Vagus Nerve. J. Physiology. 10.1113/JP282994 35524982

[B57] Penzel T. Wessel N. Riedl M. Kantelhardt J. W. Rostig S. Glos M. (2007). Cardiovascular and Respiratory Dynamics during Normal and Pathological Sleep. Chaos 17, 015116. 10.1063/1.2711282 17411273

[B58] Ponikowski P. Voors A. A. Anker S. D. Bueno H. Cleland J. G. F. Coats A. J. S. (2016). 2016 ESC Guidelines for the Diagnosis and Treatment of Acute and Chronic Heart Failure. Eur. J. Heart Fail 18, 891–975. 10.1002/ejhf.592 27207191

[B59] Raupach T. Bahr F. Herrmann P. Luethje L. Heusser K. Hasenfuss G. (2008). Slow Breathing Reduces Sympathoexcitation in COPD. Eur. Respir. J. 32, 387–392. 10.1183/09031936.00109607 18385175

[B60] Seferovic P. M. Ponikowski P. Anker S. D. Bauersachs J. Chioncel O. Cleland J. G. F. (2019). Clinical Practice Update on Heart Failure 2019: Pharmacotherapy, Procedures, Devices and Patient Management. An Expert Consensus Meeting Report of the Heart Failure Association of the European Society of Cardiology. Eur. J. Heart Fail 21, 1169–1186. 10.1002/ejhf.1531 31129923

[B61] Serrão N. F. Jr Porta A. Minatel V. Castro A. A. M. Catai A. M. Sampaio L. M. M. (2020). Complexity Analysis of Heart Rate Variability in Chronic Obstructive Pulmonary Disease: Relationship with Severity and Symptoms. Clin. Auton. Res. 30, 157–164. 10.1007/s10286-019-00659-z 31938978

[B62] Sethi S. Mahler D. A. Marcus P. Owen C. A. Yawn B. Rennard S. (2012). Inflammation in COPD: Implications for Management. Am. J. Med. 125, 1162–1170. 10.1016/j.amjmed.2012.06.024 23164484

[B63] Simonneau G. Gatzoulis M. A. Adatia I. Celermajer D. Denton C. Ghofrani A. (2013). Updated Clinical Classification of Pulmonary Hypertension. J. Am. Coll. Cardiol. 62, D34–D41. 10.1016/j.jacc.2013.10.029 24355639

[B64] Slebos D. J. Shah P. L. Herth F. J. F. Pison C. Schumann C. Hübner R. H. (2019). Safety and Adverse Events after Targeted Lung Denervation for Symptomatic Moderate to Severe Chronic Obstructive Pulmonary Disease (AIRFLOW). A Multicenter Randomized Controlled Clinical Trial. Am. J. Respir. Crit. Care Med. 200, 1477–1486. 10.1164/rccm.201903-0624OC 31404499 PMC6909835

[B65] Spaak J. Egri Z. J. Kubo T. Yu E. Ando S.-I. Kaneko Y. (2005). Muscle Sympathetic Nerve Activity during Wakefulness in Heart Failure Patients with and without Sleep Apnea. Hypertension 46, 1327–1332. 10.1161/01.hyp.0000193497.45200.66 16286569

[B66] Spiesshoefer J. Boentert M. Tuleta I. Giannoni A. Langer D. Kabitz H. J. (2019). Diaphragm Involvement in Heart Failure: Mere Consequence of Hypoperfusion or Mediated by Hf-Related Pro-inflammatory Cytokine Storms? Front. Physiol. 10, 1335–1336. 10.3389/fphys.2019.01335 31749709 PMC6842997

[B67] Spiesshoefer J. Henke C. Herkenrath S. Randerath W. Schneppe M. Young P. (2019). Electrophysiological Properties of the Human Diaphragm Assessed by Magnetic Phrenic Nerve Stimulation: Normal Values and Theoretical Considerations in Healthy Adults. J. Clin. Neurophysiol. 36, 375–384. 10.1097/WNP.0000000000000608 31145172

[B68] Spiesshoefer J. Henke C. Herkenrath S. Winfried R. Tobias B. Peter Y. (2019). Assessment of Central Drive to the Diaphragm by Twitch Interpolation: Normal Values, Theoretical Considerations, and Future Directions. Respiration 98 (4), 283–293. 10.1159/000500726 31352459

[B69] Spiesshoefer J. Herkenrath S. Mohr M. Randerath W. Tuleta I. Diller G. P. (2019). Diaphragm Function Does Not Independently Predict Exercise Intolerance in Patients with Precapillary Pulmonary Hypertension after Adjustment for Right Ventricular Function. Biosci. Rep. 39, BSR20190392. 10.1042/BSR20190392 31427479 PMC6723707

[B70] Spiesshoefer J. Becker S. Tuleta I. Mohr M. Diller G. P. Emdin M. (2019). Impact of Simulated Hyperventilation and Periodic Breathing on Sympatho-Vagal Balance and Hemodynamics in Patients with and without Heart Failure. Respiration 98, 482–494. 10.1159/000502155 31461730

[B71] Spiesshoefer J. Henke C. Herkenrath S. Brix T. Randerath W. Young P. (2019). Transdiapragmatic Pressure and Contractile Properties of the Diaphragm Following Magnetic Stimulation. Respir. Physiology Neurobiol. 266, 47–53. 10.1016/j.resp.2019.04.011 31029769

[B72] Spiesshoefer J. Henke C. Kabitz H. J. Nofer J. R. Mohr M. Evers G. (2020). Respiratory Muscle and Lung Function in Lung Allograft Recipients: Association with Exercise Intolerance. Respiration 99, 398–408. 10.1159/000507264 32403109

[B73] Spiesshoefer J. Herkenrath S. Henke C. Langenbruch L. Schneppe M. Randerath W. (2020). Evaluation of Respiratory Muscle Strength and Diaphragm Ultrasound: Normative Values, Theoretical Considerations, and Practical Recommendations. Respiration 99, 369–381. 10.1159/000506016 32396905

[B74] Spiesshoefer J. Linz D. Skobel E. Arzt M. Stadler S. Schoebel C. (2019). Sleep - the yet Underappreciated Player in Cardiovascular Diseases: A Clinical Review from the German Cardiac Society Working Group on Sleep Disordered Breathing. Eur. J. Prev. Cardiol. 28, 189–200. 10.1177/2047487319879526 33611525

[B75] Spiesshoefer J. Orwat S. Henke C. Kabitz H.-J. Katsianos S. Borrelli C. (2020). Inspiratory Muscle Dysfunction and Restrictive Lung Function Impairment in Congenital Heart Disease: Association with Immune Inflammatory Response and Exercise Intolerance. Int. J. Cardiol. 318, 45–51. 10.1016/j.ijcard.2020.06.055 32634497

[B76] Stewart A. G. Waterhouse J. C. Howard P. (1991). Cardiovascular Autonomic Nerve Function in Patients with Hypoxaemic Chronic Obstructive Pulmonary Disease. Eur. Respir. J. 4, 1207–1214. 1804668

[B77] Tamisier R. Anand A. Nieto L. M. Cunnington D. Weiss J. W. (2005). Arterial Pressure and Muscle Sympathetic Nerve Activity Are Increased after Two Hours of Sustained but Not Cyclic Hypoxia in Healthy Humans. J. Appl. Physiology 98, 343–349. 10.1152/japplphysiol.00495.2004 15448121

[B78] Vahanian A. Baumgartner H. Bax J. Butchart E. Dion R. Filippatos G. (2007). Guidelines on the Management of Valvular Heart Disease: The Task Force on the Management of Valvular Heart Disease of the European Society of Cardiology. Eur. Heart J. 28, 230–268. 10.1093/eurheartj/ehl428 17259184

[B79] Van de Borne P. Montano N. Zimmerman B. Pagani M. Somers V. K. (1997). Relationship between Repeated Measures of Hemodynamics, Muscle Sympathetic Nerve Activity, and Their Spectral Oscillations. Circulation 96, 4326–4332. 10.1161/01.cir.96.12.4326 9416900

[B80] Van De Borne P. Oren R. Abouassaly C. Anderson E. Somers V. K. (1998). Effect of Cheyne-Stokes Respiration on Muscle Sympathetic Nerve Activity in Severe Congestive Heart Failure Secondary to Ischemic or Idiopathic Dilated Cardiomyopathy. Am. J. Cardiol. 81, 432–436. 10.1016/s0002-9149(97)00936-3 9485132

[B81] van der Beek N. A. M. E. van Capelle C. I. van der Velden-van Etten K. I. Hop W. C. J. van den Berg B. Reuser A. J. J. (2011). Rate of Progression and Predictive Factors for Pulmonary Outcome in Children and Adults with Pompe Disease. Mol. Genet. Metabolism 104, 129–136. 10.1016/j.ymgme.2011.06.012 21763167

[B82] Velez-Roa S. Ciarka A. Najem B. Vachiery J.-L. Naeije R. van de Borne P. (2004). Increased Sympathetic Nerve Activity in Pulmonary Artery Hypertension. Circulation 110, 1308–1312. 10.1161/01.cir.0000140724.90898.d3 15337703

[B83] Zangrando K. Trimer R. Carvalho Jr L. C. S. Arêas G. Caruso F. Cabiddu R. (2018). Chronic Obstructive Pulmonary Disease Severity and its Association with Obstructive Sleep Apnea Syndrome: Impact on Cardiac Autonomic Modulation and Functional Capacity. Copd 13, 1343–1351. 10.2147/copd.s156168 PMC5927062 29731622

